# Efficacy of SMART Stent Placement for Salvage Angioplasty in Hemodialysis Patients with Recurrent Vascular Access Stenosis

**DOI:** 10.4061/2011/464735

**Published:** 2011-11-20

**Authors:** Shingo Hatakeyama, Terumasa Toikawa, Akiko Okamoto, Hayato Yamamoto, Kengo Imanishi, Teppei Okamoto, Noriko Tokui, Yuichiro Suzuki, Naoki Sugiyama, Atsushi Imai, Yasuhiro Hashimoto, Shigemasa Kudo, Takahiro Yoneyama, Takuya Koie, Noritaka Kamimura, Hisao Saitoh, Tomihisa Funyu, Chikara Ohyama

**Affiliations:** ^1^Department of Advanced Transplant and Regenerative Medicine, Hirosaki University Graduate School of Medicine, Hirosaki 036-8562, Japan; ^2^Department of Radiological Technology, Oyokyo Kidney Research Institute, Hirosaki 036-8243, Japan; ^3^Department of Urology, Oyokyo Kidney Research Institute, Hirosaki 036-8243, Japan; ^4^Department of Urology, Hirosaki University Graduate School of Medicine, Hirosaki 036-8562, Japan

## Abstract

Vascular access stenosis is a major complication in hemodialysis patients. We prospectively observed 50 patients in whom 50 nitinol shape-memory alloy-recoverable technology (SMART) stents were used as salvage therapy for recurrent peripheral venous stenosis. Twenty-five stents each were deployed in native arteriovenous fistula (AVF) and synthetic arteriovenous polyurethane graft (AVG) cases. Vascular access patency rates were calculated by Kaplan-Meier analysis. The primary patency rates in AVF *versus* AVG at 3, 6, and 12 months were 80.3% *versus* 75.6%, 64.9% *versus* 28.3%, and 32.3% *versus* 18.9%, respectively. The secondary patency rates in AVF *versus* AVG at 3, 6, and 12 months were 88.5% *versus* 75.5%, 82.6% *versus* 61.8%, and 74.4% *versus* 61.8%, respectively. Although there were no statistically significant difference in patency between AVF and AVG, AVG showed poor tendency in primary and secondary patency. The usefulness of SMART stents was limited in a short period of time in hemodialysis patients with recurrent vascular access stenosis.

## 1. Introduction

Although percutaneous transluminal angioplasty (PTA) is the standard for treatment of vascular access venous stenosis and occlusion since the 1980s [1], it carries a high rate of restenosis, and repeated endovascular intervention is often necessary. Compared with native arteriovenous fistula (AVF), synthetic arteriovenous grafts (AVG) are associated with much higher rates of failure and intervention because of the increased rates of stenosis/thrombosis. The intervention rates for AVG are reported to be about five times higher than those for AVF, with patency rates less than 50% at 3 years [2]. To improve these statistics, various indications for the use of endovascular stents have been studied since 1989 [3], including elastic recoil [4–6], rapid recurrence [7], and venous rupture after PTA [8].

Although endovascular stent placement is one of the standard treatments in percutaneous coronary and peripheral artery disease, its role in the treatment of vascular access stenosis remains controversial. Early studies reported that routine use of metallic stents failed to provide any additional benefit when compared with PTA alone [3]. Multiple studies have compared endovascular stents to PTA in terms of patency, but most of references reported limited or no advantages to a stent placement for peripheral venous and graft stenoses [2, 4, 9–13]. Cohort study conducted by Vogel and Parise reported that nitinol shape-memory alloy-recoverable technology (SMART) stents improved primary and secondary graft patency in AVG cases [14]. Recent publications have reported that covered stents or stent-graft placement was not inferior in patency to PTA alone [15–17]. Therefore, the exact role and indications of stent placement in the treatment of stenotic lesions in AVF and AVG remain unclear.

In this study, we prospectively observed 50 patients in whom 50 SMART stents were used as salvage therapy for recurrent peripheral venous stenosis and compared patency between AVF and AVG in cases of post-PTA failure caused by elastic recoil and rapidly recurrent stenosis.

## 2. Patients and Methods

### 2.1. Study Population

This study was approved by the institutional ethical committee of Oyokyo Kidney Research Institute. Informed consent was obtained from all patients. From June 2009 to September 2010, 548 patients underwent hemodialysis at the Oyokyo Kidney Research Institute in Hirosaki, Japan. During that period, total 453 PTAs were performed and all endovascular procedures were performed by the well-trained interventional urologists in this institute. We followed up consecutive 50 patients who underwent SMART stent placement as salvage therapy for recurrent peripheral venous stenosis. Twenty-five stents each were deployed in native AVF and synthetic AVG.

### 2.2. Inclusion and Exclusion Criteria

Inclusion criteria were (1) age of 18 to 90 yr and a hemodialysis access consisting of an AVF or an AVG located in the arm, (2) stable hemodialysis sessions were performed, (3) color Doppler ultrasonography or angiographic evidence of 1 or more stenotic lesion, 7 cm or less in length, and 50% or more stenosis compared with previous evaluation, (4) difficult vascular access; percutaneous endovascular therapy was thought to be the best treatment choice for the identified lesion because it is difficult to develop new vascular accesses in other lesions, (5) recoil and/or kinked venous stenosis within the past 3 months, (6) more than 3 time of PTA history, (7) Eastern Cooperative Oncology Group Performance Status (ECOG PS) [18] grade 0 to 4. In this study, poor general health (ECOG PS > 2), major concomitant disease (e.g., terminal cancer), or other medical condition likely to result in death within 6 months after the time of implantation were not in exclusion criteria. Exclusion criteria were (1) recurrent stenosis with a corresponding thrombosis treated within 7 days before enrollment, (2) a blood coagulation disorder or sepsis, (3) a contraindication to the use of contrast medium, (4) infected arteriovenous access graft, (5) presence of an alternate stent, (6) stenotic lesion which needed more than 6 mm diameter stent in upper-arm outflow vein or subclavian vein, and (7) occluded vascular access.

### 2.3. Technical Description

AVF and AVG were initially cannulated with a 16–18-gauge puncture needle, and a 6-French catheter sheath was inserted over a guide wire into the lumen of AVF or AVG. Diluted contrast media was injected for digital subtraction angiography to localize stenotic lesions. An angioplasty balloon (Conquest, 6 mm in diameter, 25–30 atmospheres in pressure; BARD Inc., Murray Hill, NJ, USA or LUMEFA, 6 mm in diameter, 18 atmospheres in pressure; Toray Medical Co., Chiba, Japan) was placed and inflated at the level of the stenotic site. If severe elastic recoil or significant residual stenosis was observed or if the stenosis had recurred shortly after a previous intervention, a SMART stent (SMART Control; Cordis/Johnson & Johnson, Warren, NJ, USA) that was 6 mm in diameter and 40–80 mm in length was deployed. All patients received 3000–4000 U of heparin during the procedure. Antiplatelet agents remained unchanged in all patients before and after the intervention.

### 2.4. Follow-Up Protocol and Intervention Indications

For blood access followup, peripheral vascular stenosis was monitored by color Doppler ultrasonography every 1–3 months. Catheter-based interventions were performed in patients who met the clinical criteria for vascular access dysfunction and had stenosis of more than 50%.

### 2.5. Evaluation

Primary (unassisted) and secondary (assisted) patency was calculated from the date of stent placement to the first subsequent intervention and permanent blood access failure. Primary and secondary patency rates were calculated using the Kaplan-Meier method and log-rank test.

### 2.6. Statistical Analysis

Statistical analyses were performed using SPSS (SPSS Inc., ver. 12.0, Chicago, IL, USA) and Microsoft Excel (Microsoft Co., Redmond, WA, USA) programs. All values included in the figures and text are expressed as means ± SD. Datasets were compared using the Mann-Whitney's *U* test or a paired *t *test. A *P* value of less than 0.05 was considered to be significant.

## 3. Results

Fifty SMART stents were deployed in cases of dysfunctional blood access for salvage therapy in 50 patients. The median number of follow-up days after SMART stent deployment was 290. Patient characteristics are shown in Table 1. Twenty-five SMART stents each were deployed in AVF and AVG cases. No difference was observed in patient backgrounds. Stent location was significantly different between AVF and AVG cases because major stenotic lesions were located in the lower arm in AVF cases and in the upper arm in AVG cases.

**Figure 1:**
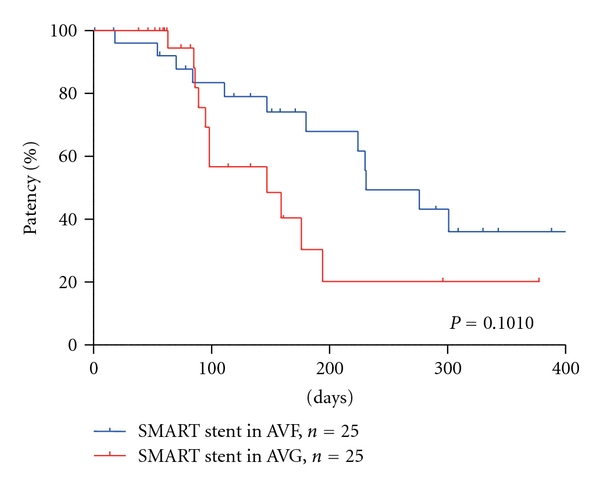
Primary patency rates in AVF and AVG. No significant difference was observed in the primary patency rates between AVF and AVG, but patency in AVG showed inferior to AVF.

To determine the efficacy of SMART stent placement for AVF and AVG, primary patency rates were evaluated using the Kaplan-Meier method. The primary patency ratios for AVF *versus* AVG at 3, 6, and 12 months were 80.3% *versus *75.6%, 64.9% *versus* 28.3%, and 32.3% *versus* 18.9%, respectively (Table 2). The 50% access patency times were 230 days in AVF cases and 133 days in AVG cases. There was no significant difference between the efficacy of SMART stent placement in AVF and AVG cases (*P* = 0.1010), but inferior in AVG at 6 and 12 months (64.9% *versus* 28.3% and 32.3% *versus* 18.9%, resp.,) (Table 2, Figure 1).

**Figure 2:**
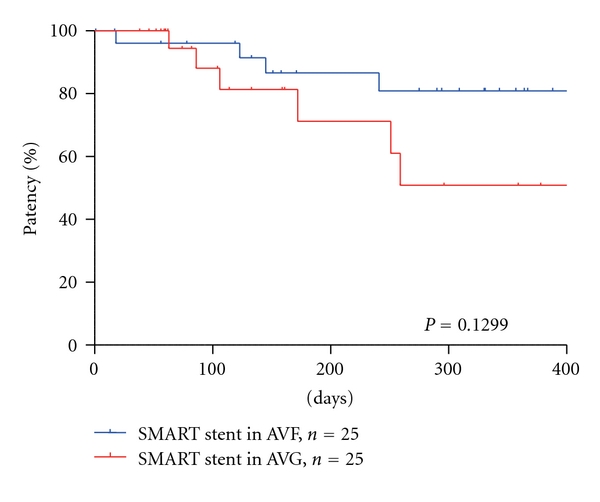
Secondary patency rates of SMART stent placement in AVF and AVG. There was no significant difference in the secondary patency rates between AVF and AVG, but patency in AVG showed inferior to AVF.

 The secondary patency rates for SMART stents at 3, 6, and 12 months were 83.4%, 74.2%, and 68.0%, respectively (Table 3). Restenosis after stent placement occurred in 23 patients (46%). The reasons for primary patency failure were in-stent stenosis (14/23, 61%), outflow stenosis (4/23, 17%), and stenosis unrelated to stent placement (5/23, 22%). The ratio of outflow stenosis onset was significantly higher in AVF cases (*P* = 0.045). The secondary patency ratios of patients with SMART stents did not significantly differ between AVF and AVG (*P* = 0.1299), but inferior in AVG at 3, 6, and 12 month (88.5% *versus* 75.5%, 82.6% *versus* 61.8%, and 74.4% *versus* 61.8%, resp.,) (Table 3, Figure 2).

 Complications associated with SMART stent deployment were not observed. Episodes of symptomatic arterial embolization of thrombus, signs of pulmonary embolism, or stent infection during followup were not observed.

## 4. Discussion

The maintenance of vascular access in hemodialysis patients is critical to the quality of life and survival. However, high rates of restenosis and repeat endovascular intervention associated with PTA remain problematic issues. Multiple devices and techniques such as cutting balloon angioplasty [19], metallic stents [12, 14], and more recently stent grafts or covered stents [15] have been used. Metallic stents have been used for blood access for more than 20 years [20], but the effect of metallic stents in peripheral vascular access is still controversial. Although there is some evidence that metallic stents have the potential to alleviate rapidly recurring peripheral venous stenosis [7], use of metallic stents (Wallstent) was not recommended because a prospective, randomized trial showed no advantage of its use over conventional angioplasty [11].

 On the other hand, some studies supported the efficacy of metallic stent (SMART) placement for salvage angioplasty. A SMART stent is composed of nitinol, a nickel-titanium metallic alloy with shape memory function. The advantages of a SMART stent over conventional metallic stents (Wallstent) include a high degree of strength and superelasticity. Nitinol possesses a high degree of flexibility and kink and fatigue resistance; these properties are not affected by repeated interventions. In addition, its superelasticity ensures equal distribution of wall contact and confers the ability to adapt to native vessel contours more successfully than conventional stents. Vogel and Parise reported improved performance using the nitinol stent, demonstrating the efficacy of SMART stent placement in retrospective [12] and prospective, nonrandomized studies [14] for dysfunctional AVG salvage therapy. In previous reports, the 3-, 6-, and 12-month patency rates of metallic stents were 77–88%, 51–67%, and 20–41%, respectively (Table 4).

 Our results showed that there was no statistical different in patency of AVF and AVG, but AVG showed poor tendency in primary and secondary patency. Incidents of out-flow stenosis were significantly higher in AVF, and in-stent stenosis were significantly higher in AVG. Out-flow stenoses in AVF might be caused by longer out-flow vein of forearm AVF, secondary to hemodynamic change or selection bias because of the small number of patients. In-stent stenoses in AVG were mainly caused by ingrowth of neointimal hyperplastic tissue through the mesh of the metallic stent at the venous-graft anastomotic site. These data suggest a prevention of in-growth tissue in SMART stent has potential to improve poor patency in AVG. To further improve patency and reduce the incidence of luminal hyperplasia, several authors have explored the use of the stent-graft, which is a self-expanding nitinol stent covered in carbon-impregnated expanded polytetrafluoroethylene. The use of stent-grafts or covered stents appears to be logical to prevent ingrowth of neointimal hyperplastic tissue. Haskal et al. [15] performed a randomized, prospective, multicenter trial involving 190 patients and clearly indicated noninferiority extending the patency of AVG cases at 6 months. Further studies are necessary to determine appropriate indications for the use of stents in AVG with rapid recurrence.

 In this present study, salvage SMART stent placement provides similar primary patency in previous reports [12, 14, 21–24] (Table 4). The main reason of insufficient patency was that the patients in this study were selected because they were basically PTA failures with either elastic lesions or rapid recurrences. Therefore, a use of SMART stent is only in selective patients, in whom without stent placement, the vascular access will be restenotic or abandoned immediately or in a very short time. But after stent placement, the patency may be the same or only slightly better than that of regular cases [4, 10, 11]. These results suggest that there were no or limited clinical advantage of routine use of bare metallic stent over angioplasty only, although stent graft had revealed some clinical benefits. However, our results have certain limitations such as small sample size, a nonrandomized trail performed at a single institute, without appropriate control group. Large randomized, comparative studies are necessary to confirm the usefulness and appropriate indications stent placement in hemodialysis patients.

## 5. Conclusion

SMART stent placement was a safe for salvage angioplasty in treating recurrent peripheral vascular stenosis, but the usefulness of SMART stents was limited in a short period of time despite our efforts to improve patency. Larger studies are required to determine appropriate indications.

## Figures and Tables

**Table 1:** Patient characteristics. Twenty-five SMART stents each were deployed in AVF and AVG cases. There was no significant difference, other than stent location, between the backgrounds of the patients in the two groups.

	ALL	AVF	AVG	*P* value
Number of patients	50	25	25	
Number of stents	50	25	25	
Age	71.6 ± 11.3	71.2 ± 11.3	72.2 ± 11.6	n.s.
Gender (M/F)	24/27	13/12	10/15	n.s.
Primary renal disease				
DM	24	12	12	n.s.
non DM	26	13	13	
Hemodialysis history (years)	7.3 ± 6.1	7.8 ± 6.2	6.6 ± 5.9	n.s.
PTA history (times)	4.6 ± 3.9	4.5 ± 3.5	4.8 ± 4.3	n.s.
Difficult vascular access*	50	25	25	
Poor general health** (%)	35 (70%)	18 (72%)	17 (68%)	n.s.
Use of antiplatelet agents	49	24	25	n.s.
Stent location				
Upper arm	22	4	18	0.0002
Lower arm	28	21	7	

*Difficult vascular access; percutaneous endovascular therapy thought to have been the best treatment choice for the identified lesion because it is difficult to develop new vascular accesses in other lesions. **Poor general health; Eastern Cooperative Oncology Group Performance Status grade 3 or 4.

**Table 2:** Primary patency for SMART stent placement in AVF and AVG cases. There was no significant difference in primary patency rates between AVF and AVG cases, but patency in AVG showed inferior to AVF.

	All	AVF	AVG	*P* value
*n*	50	25	25	
Primary patency (Days)	140 ± 105	168 ± 118	110 ± 78.9	0.0051
(range)	(17–401)	(17–401)	(38–378)	
Primary patency (%)				
3 months	79	80.3	75.6	0.1010
6 months	51.3	64.9	28.3	
12 months	27.1	32.3	18.9	

**Table 3:** Secondary patency for SMART stent placement in AVF and AVG cases.There was no significant difference in secondary patency rates between AVF and AVG cases, but patency in AVG showed inferior to AVF. The rate of outflow stenosis onset was significantly higher in AVF cases (*P* = 0.045).

	All	AVF	AVG	*P* value
*n*	50	25	25	
Secondary patency (Days)	189 ± 129	224 ± 129	151 ± 121	n.s.
(range)	(18–259)	(18–241)	(63–259)	
Secondary patency (%)				
3 months	83.4	88.5	75.5	
6 months	74.2	82.6	61.8	0.1299
12 months	68	74.4	61.8	
Reasons of primary patency failure				
In-stent stenosis (%)	14 (28)	6 (24)	8 (32)	n.s.
Out-flow stenosis (%)	4 (8)	4 (16)	0 (0)	0.045
Others (%)	5 (10)	2 (8)	3 (12)	n.s.

**Table 4:** Summary of recent reports of outcome using metallic stents. The 3-, 6-, and 12-month patency rates were 77–88%, 51–67%, and 20–41%, respectively.

Investigators	Year	*n*	Study design	Stent type	AVF or AVG	Primary patency (%)			(months)
						3 M	6 M	12 M	
Vogel and Parise [12]	2004	53	Retrospective	SMART	AVG	77 (61–93)	51 (34–67)	20 (12–27)	mean 8.9
Vogel and Parise [14]	2005	25	Prospective, Non-randomized	SMART	AVG	88 (75–100)	67 (48–86)	41 (21–61)	mean 8.2
Pan et al. [21]	2005	12	Retrospective	Wallstent, Jostent	AVF	92 ± 8	81 ± 12	31 ± 17	n/a
Liang et al. [22]	2006	23	Observational	Wallstent, nitinol	AVG	69 ± 9	41 ± 10	30 ± 10	n/a
Maya and Allon [23]	2006	14	Prospective, Non-randomized	Wallstent, SMART, Protégé, Fluency	AVG	48	19	n/a	median 2.8
Chan, M.R. et al. [24]	2008	211	Retrospective	SMART	AVG	69	25	n/a	median 4.4
Current study	2011	50	Prospective, Observational	SMART	Both	79 ± 9	51 ± 15	27 ± 16	median 3.8
		25			AVF	80 ± 10	65 ± 16	32 ± 21	median 5.2
		25			AVG	76 ± 15	28 ± 22	19 ± 17	median 2.9

## Abbreviation

PTA: Percutaneous transluminal angioplasty,
SMART: Shape memory alloy recoverable technology
AVF: Arteriovenous fistula
AVG: Arteriovenous graft
DM: Diabetes mellitus.
